# Furosine Induced Apoptosis by the Regulation of STAT1/STAT2 and UBA7/UBE2L6 Genes in HepG2 Cells

**DOI:** 10.3390/ijms19061629

**Published:** 2018-05-31

**Authors:** Huiying Li, Lei Xing, Nan Zhao, Jiaqi Wang, Nan Zheng

**Affiliations:** 1 Institute of Animal Sciences, Chinese Academy of Agricultural Sciences, Beijing 100193, China; thufit2012@126.com (H.L.); lei_xing163@163.com (L.X.); wang-jia-qi@263.net (J.W.); 2 College of Animal Science and Technology, Anhui Agricultural University, Hefei 230036, China; qingnan0705@163.com

**Keywords:** furosine, STAT1, STAT2, UBA7, UBE2L6, apoptosis

## Abstract

As a typical product in the Miallard reaction, research on the quantitative detection of furosine is abundant, while its bioactivities and toxic effects are still unclear. Our own work recently demonstrated the induction of furosine on apoptosis in *HepG2* cells, while the related mechanism remained elusive. In this study, the effects of furosine on cell viability and apoptosis were detected to select the proper dosage, and transcriptomics detection and data analysis were performed to screen out the special genes. Additionally, SiRNA fragments of the selected genes were designed and transfected into *HepG2* cells to validate the role of these genes in inducing apoptosis. Results showed that furosine inhibited cell viability and induced cell apoptosis in a dose-dependent manner, as well as activated expressions of the selected genes *STAT1* (signal transducer and activator of transcription 1), *STAT2* (signal transducer and activator of transcription 2)*, UBA7* (ubiquitin-like modifier activating enzyme 7), and *UBE2L6* (ubiquitin-conjugating enzyme E2L6), which significantly affected downstream apoptosis factors *Caspase-3* (cysteinyl aspartate specific proteinase-3)*, Bcl-2* (B-cell lymphoma gene-2), *Bax* (BCL2-Associated gene X), and *Caspase-9* (cysteinyl aspartate specific proteinase-9). For the first time, we revealed furosine induced apoptosis through two transcriptional regulators (*STAT1* and *STAT2*) and two ubiquitination-related enzymes (*UBA7* and *UBE2L6*).

## 1. Introduction

The Maillard reaction is a classical reaction resulting from the food heating process. As it results in the covalent attachment of sugars and the degradative products to lysine and arginine residues, the generated amadori products are known as Maillard reaction products (MRPs) [1,2]. As one of the typical MRPs in the Miallard reaction, furosine (C_12_H_18_N_2_O_4_, Mw 254.28) exists in lots of food items including dairy products, infant formula, cereals, honey, and bakery products, etc. [3,4,5]. Furosine has been proven to be a stable derivative of the amadori compound, which is also well-known as a reliable marker and indicator of the nutritional evaluation of the heat treatment of food [6,7]. 

Articles on the quantitative detection and analysis of furosine are abundant, while research about the toxicity evaluation and mechanism exploration of furosine are rare. Until now, there has been no available data providing evidence for metabolic courses exploration of furosine, though safety evaluation and limit control of furosine in food products have become necessary and pressing. In our recent research, furosine was found to pose a toxic effect on liver tissue through activating downstream apoptosis and an inflammatory reaction, embodied in up-regulation of *Caspase-3*, *Bax, Bcl-2*, *IL-1β,* and *TNF-α* [8]. However, the related molecular mechanism of furosine still remained unclear. In this study, we exerted *HepG2* cell transcriptomics detection to screen out the special genes, *STAT1*, *STAT2*, *UBA7*, and *UBE2L6*, which might play a key role in participating cell apoptosis induced by furosine. Then, we investigated the effect of these selected genes on downstream apoptosis factors, in order to further validate furosine induced cell apoptosis through the two transcriptional regulators (*STAT1* and *STAT2*) and two ubiquitination-related enzymes (*UBA7* and *UBE2L6*).

## 2. Results

### 2.1. Furosine Inhibited HepG2 Cell Viability

**Figure 1:**
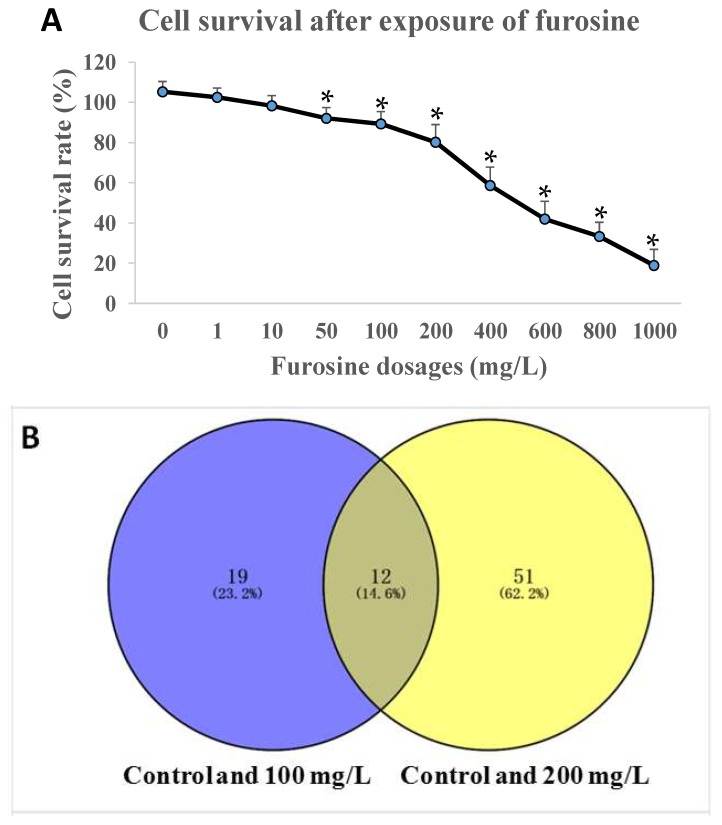

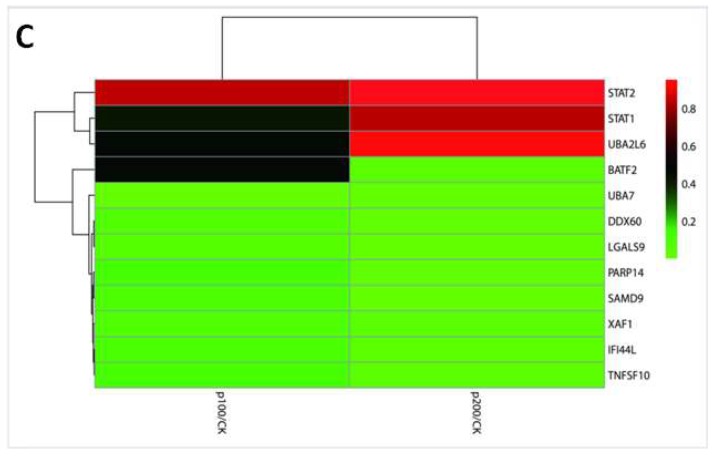
*HepG2* cell viability and transcriptomics detection. (**A**) Furosine inhibited cell survival rate with a dose-effect relationship. The data was represented as mean ± SD (standard deviation), * *p* < 0.05, compared with the control (*n* = 8); (**B**) Overlapping of selected genes with changed expressions in control, 100 mg/L group, and 200 mg/L group, through cell transcriptomics detection (*n* = 3); (**C**) Heatmap of 12 special genes.

To test the effect of furosine on *HepG2* cell viability, different dosages (0, 1, 10, 50, 100, 200, 400, 600, 800 and 1000 mg/L) of furosine were applied utilizing the CCK8 kit. Reduction in cell viability was observed after being exposed to furosine with a dosage of 100 mg/L or above (*p* < 0.05) for 48 h, with a dose-effect relationship (Figure 1A). Considering that the cell viability at the dosages of 100 and 200 mg/L were 90.3 ± 9.6% and 83.5 ± 5.0%, respectively, the two dosages were selected as the appropriate ones in the following experiments.

### 2.2. Transcriptomics Detection of HepG2 Cells 

The transcriptomics strategy was used in *HepG2* cells before or after 100/200 mg/L furosine treatment to detect the important genes participating in the signal pathway, and the number of biological replicates in each group was three (*n* = 3). We found that 31 gene expressions changed in the control group vs. 100 mg/L group condition, while 63 gene expressions changed in the control vs. 200 mg/L condition. Expressions of 19 genes or 51 genes were changed only in control vs. 100 mg/L or control vs. 200 mg/L conditions. Together, there were 12 genes with a similar expression pattern between the control vs. 100 mg/L and control vs. 200 mg/L, including *STAT1*, *STAT2*, *UBA7*, and *UBE2L6*, etc. (Figure 1B, Venn diagram). The expression levels of the 12 special genes were integrated into the heatmap (Figure 1C).

### 2.3. The Effect of Furosine on Cellular Location of STAT1, STAT2, UBA7, and UBE2L6

The expressions and subcellular locations of *STAT1/2*, *UBA7*, and *UBE2L6* were also investigated by in situ fluorescence imaging using confocal. The GFP-labeled of these factors fused proteins were transfected into *HepG2*, and green fluorescence indicates that the fused proteins all expressed successfully. As the time exposure to furosine increased, the green light of the factors were boosted, indicating that the expressions were enhanced. To exactly locate the protein, the PI dye was used to stain the compartment of the nucleus.

**Figure 2:**
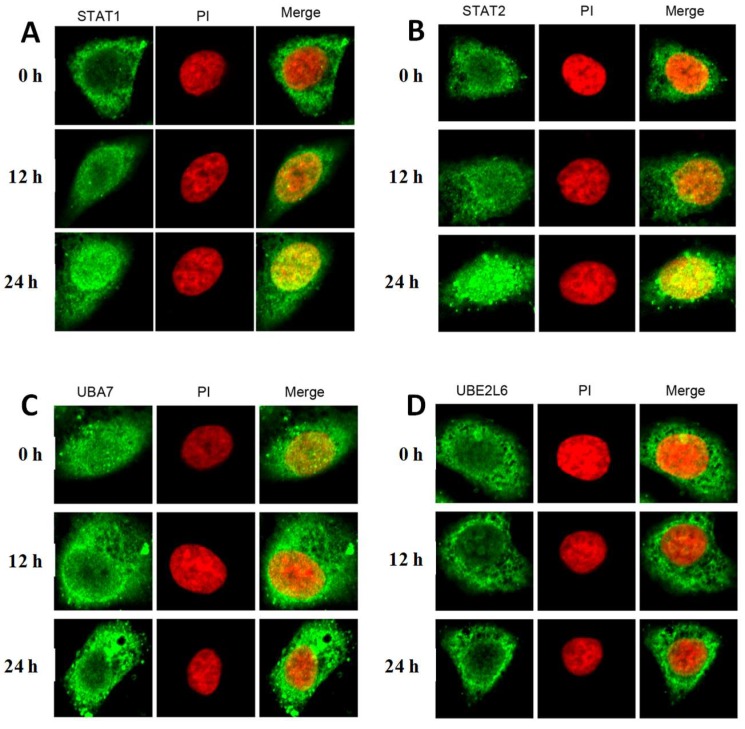
Translocation detection of four factors in *HepG2* cells treated by furosine (100 mg/L) by confocal. (**A**) *STAT1* in cell; (**B**) *STAT2* in cell; (**C**) *UBA7* in cell; (**D**) *UBE2L6* in cell. Green light stands for proteins of interest in the cell, and red light stands for the nucleus. The pictures were captured at 100× magnification.

As the merged results of these proteins and nucleus showed, *STAT1* and *STAT2* were found to be mainly located in the cytoplasm of normal *HepG2* cells, and they began to translocate into the nucleus with the treatment of furosine (Figure 2A,B). *UBA7* was found to locate in both cytosol and the nucleus under a normal condition, and most of the *UBA7* protein in furosine-treated cells translocated into cell cytosol (Figure 2C). Most of the *UBE2L6* protein located in cell cytosol without outside stimulation, and it seemed to translocate into cytosol in furosine-treated cells (Figure 2D). 

### 2.4. The Effect of Furosine on mRNA and Protein Expression of STAT1, STAT2, UBA7, and UBE2L6

**Figure 3:**
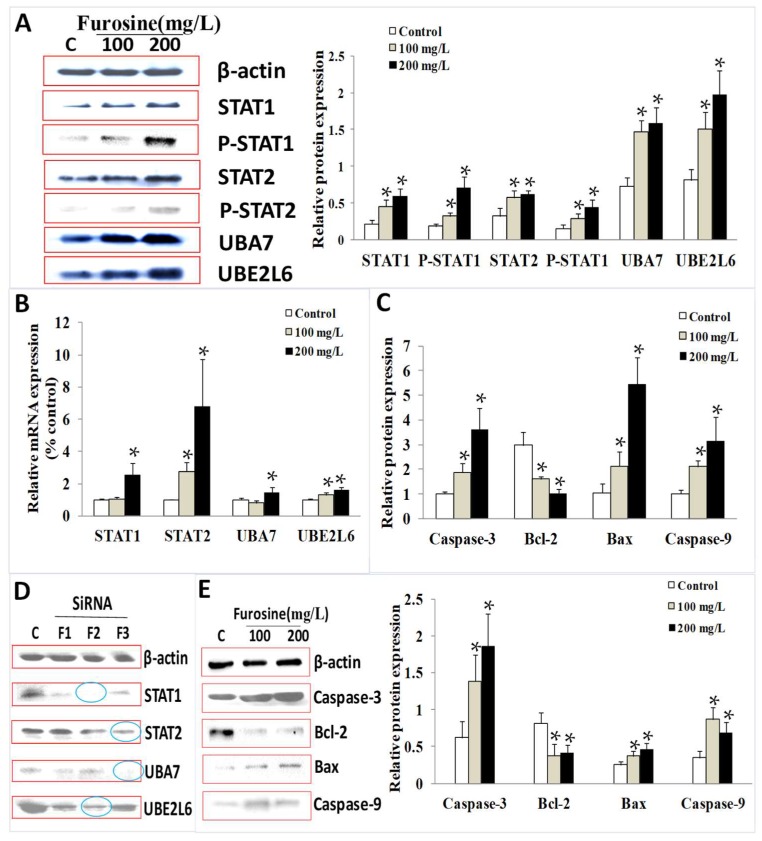
mRNA level and protein level of *STAT1/2*, *P-STAT1/2*, *UBA7*, *UBE2L6*, *Caspase-3*, *Bcl-2*, *Bax*, and *Caspase-9* affected by furosine. (**A**) Furosine up-regulated expressions of *STAT1/2*, *P-STAT1/2*, *UBA7*, and *UBE2L6* at protein level; (**B**) Furosine up-regulated expressions of *STAT1/2*, *UBA7*, and *UBE2L6* at mRNA level; (**C**) Furosine up-regulated expressions of *Caspase-3/9*, *Bax*, and *Bcl-2* at protein level; (**D**) Selection of high-efficient SiRNA fragment in *HepG2* cells. Blue circle stands for the high-effeciency SiRNA fragments of each factor which would be chosen and utilized in Figure 4 and Figure 5; (**E**) Furosine up-regulated expressions of *Caspase-3/9*, *Bax*, and *Bcl-2* at mRNA level. The values of biochemical indicators were represented as mean ± SD, * *p* < 0.05, compared with the control (*n* = 3).

To validate the effect of furosine on the expression of these factors, in terms of both the mRNA level and protein level, qPCR and western blotting detections were performed. Results showed that expressions of *STAT1*, *STAT2*, *P-STAT1*, *P-STAT2*, *UBA7*, and *UBE2L6* proteins in the whole *HepG2* cells increased significantly (*p* < 0.05) with the treatment of furosine. Additionally, mRNA expressions of *STAT1*, *STAT2*, *P-STAT1*, *P-STAT2*, *UBA7*, and *UBE2L6* in the whole cells increased significantly (*p* < 0.05) with the treatment of furosine, in a dose-dependent manner, when compared with the control (Figure 3A,B).

### 2.5. The Role of Furosine in Inducing Cell Apoptosis

To investigate the role of furosine in regulating the apoptosis-related pathway, downstream factors including *Caspase-3*, *Bcl-2*, *Bax*, and *Caspase-9* were detected by qPCR (quantitative real time polymerase chain reaction) and western blotting. Results showed that when compared with the control, *Caspase-3*, *Bax*, and *Caspase-9* were found to be significantly up-regulated in furosine treatment groups (*p* < 0.05), and the inhibitor of apoptosis *Bcl-2* decreased significantly (*p* < 0.05), in terms of both the mRNA level (Figure 3C) and protein level (Figure 3E). Moreover, expressions of these factors were found to be changed in furosine treatment groups in a dosage-dependent manner. 

### 2.6. The Role of STAT1, STAT2, UBA, and UBE2L6 in Participating in Apoptosis Induced by Furosine

To estimate the function of these factors in the furosine-induced signal pathway, the different expression patterns of regulatory factors were investigated in *HepG2* cells and SiRNA-treated *HepG2* cells. The gene fragments for SiRNA of *STAT1/2*, *UBA7*, and *UBE2L6* were designed and used to test the efficiency of gene silence, and the gene fragments of high efficiency were screened out for further study (blue arrows, in Figure 3D). 

**Figure 4:**
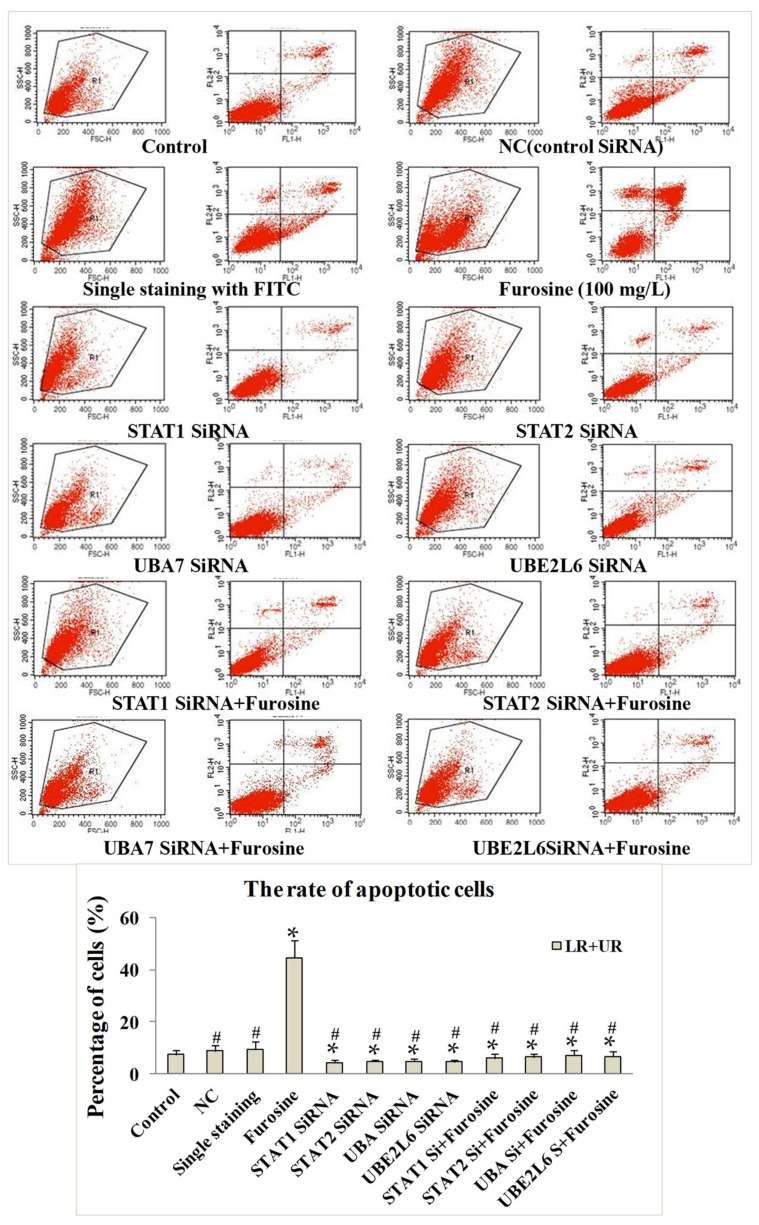
The effect of furosine on cell apoptosis by annexin V/PI staining. Control stood for cells without any treatment; NC stood for cells treated with control SiRNA; single staining group stood for cells stained with annexin-FITC; Si stood for SiRNA of the aimed factor. All the data were represented as mean ± SD, * *p* < 0.05, compared with the control. # *p* < 0.05, compared with the furosine treatment group (*n* = 3).

To investigate the effect of furosine on cell apoptosis, an annexin V/PI (propidium iodide) staining kit was utilized. Results showed that the cell apoptosis rates in the NC (negative control) group and single staining group seemed to exhibit no obvious change compared to the control. When we treated the cells with *STAT1*/*STAT2*/*UBA7*/*UBE2L6* SiRNAs, respectively, the apoptosis rates were lower than the control/ NC group/ single staining group (*p* < 0.05). Additionally, treatment of furosine significantly induced the apoptosis of *HepG2* cells when compared with the control (*p* < 0.05). When we treated the cells with SiRNAs of *STAT1*/*STAT2*/*UBA7*/*UBE2L6* + furosine (100 mg/L), there seemed to be no obvioius up-regulation of apoptosis rates compared with the *STAT1*/*STAT2*/*UBA7*/*UBE2L6* SiRNAs groups. The above results indicated that the four factors played key roles in inducing and participating in *HepG2* cell apoptosis (Figure 4).

**Figure 5:**
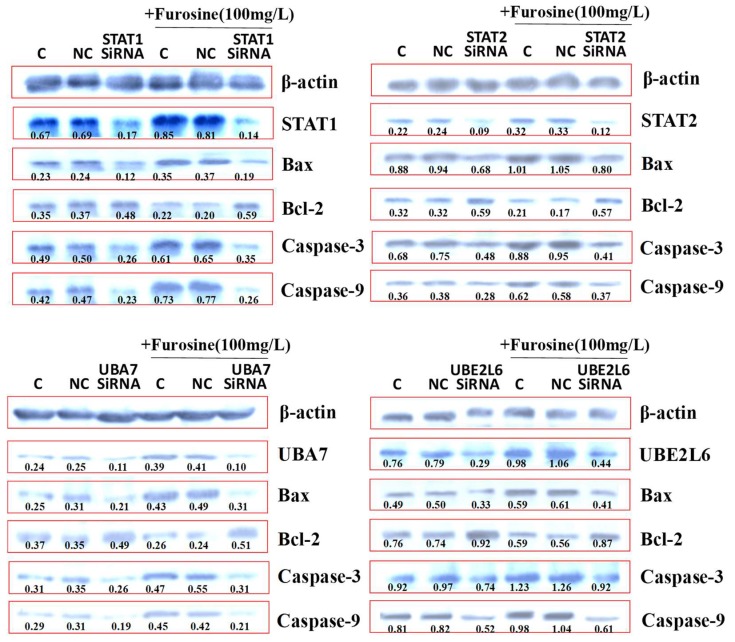
Proteins expression of *STAT1/2*, *UBA7*, *UBE2L6*, *Caspase-3*, *Bcl-2*, *Bax*, and *Caspase-9* treated with of SiRNA and furosine. The values of biochemical indicators were represented as mean. C stands for control, and NC stands for group treated with control SiRNA.

In the *STAT1*-SiRNA+furosine group, expression of *Caspase-3* decreased compared with the *STAT1*-SiRNA group (*p* < 0.05), while the other genes exhibited a similar expression pattern to the *STAT1*-SiRNA group. In the *STAT2*-SiRNA+furosine group, expressions of *Caspase-9*, *Bax*, and *Bcl-2* were comparable to those in the *STAT2*-SiRNA group. In the *UBA7*-SiRNA+furosine group, expressions of *Bax* and *Caspase-9* were down-regulated, whereas *Bcl-2* exhibited a higher expression, compared to those in the *UBA7*-SiRNA group (*p* < 0.05). In the *UBE2L6*-SiRNA+furosine group, expressions of *Bax* and *Caspase-9* decreased more than those in the *UBE2L6*-SiRNA group (*p* < 0.05) (Figure 5).

## 3. Discussion

*STAT1* and *STAT2* are transcription factors belonging to the *STAT* protein family. *STAT* molecules can be phosphorylated by kinase-associated receptors and activated by dimerization and forming homodimers or heterodimers, which then translocate into the nucleus as transcription factors. As the most classical members of the *STATs* family, *STAT1* and *STAT2* were found to be activated by several inflammatory ligands including interferon gamma (*IFN-γ*), interferon alpha (*IFN-f*), epidermal Growth Factor (*EGF*), and tumor necrosis factor alpha (*TNF-α*), etc. [9]. *STAT1* and *STAT2* are involved in cell viability, proliferation, apoptosis, and the cell cycle, which mediate inflammation, oxidation, tumors, and organ injury through the *STAT1*/*STAT2*-related pathway [10,11,12,13,14,15,16].

*UBA7* and *UBE2L6* belong to ubiquitination-related enzymes. The ubiquitin-modified proteins are always abnormal or short-lived proteins, which are finally degradated. Ubiquitination course is usually considered to involve three classes of enzymes: ubiquitin-activating enzymes (E1s), ubiquitin-conjugating enzymes (E2s), and ubiquitin-protein ligases (E3s). *UBA7* is regarded as one kind of E1 ubiquitin-activating enzyme, and *UBE2L6* is regarded as a member of the E2 ubiquitin-conjugating enzyme group. The two enzymes are involved in protein metabolization like proteasomal degradation and cell apoptosis, which are tightly related to inflammation, organ injury, virus infection, and tumors, etc. [17,18,19,20,21,22,23,24]. 

There are several articles elucidating the relationship between *STATs* and ubiquitin-related enzymes, especially in cell apoptosis activated by hypoxia, inflammation, and tumors. Research has proved that *STAT1/2* can induce cell apoptosis and protein degradation, requiring ubiquitin-related enzymes including *UBA7* and *UBE2L6* [25,26,27,28]. Considering the above reuslts, we selected the four factors through transcriptomics detection and tested their translocation and expression levels in furosine treatment cells. 

IF results showed that the fluorescence signal of *STAT1* and *STAT2* in the cell nucleus increased, the fluorescence signal of *UBA7* in the cell nucleus weakened, and the one of *UBE2L6* seemed to exhibit no obvious change, when comparing them with the control. The above results could be explained as follows: As key transcriptional regulators, *STAT1* and *STAT2* entered into the cell nucleus with the aim of regulating the expression of several apoptotic proteins after the stimulation of furosine; *UBA7* usually locates in the whole cell, and it tended to translocate into cell cytosol and degradate proteins in apoptosis course in the furosine treatment group; *UBE2L6* always locates in cell cytosol and still participates in protein degradation in cytosol, so its location seemed to display no change with the treatment of furosine [29,30,31,32,33,34]. The above results suggested that furosine activated the four factors in *HepG2* cells, embodying the translocation of these factors in *HepG2* cells.

Combined with the results of q-PCR detection and western blotting detection, the mRNA levels of the four factors (*STAT1/2*, *UBA7*, *UBE2L6*) in the furosine treatment groups were expressed significantly higher than the control, and the protein levels of the six factors (*STAT1/2*, *P-STAT1/2*, *UBA7*, *UBE2L6*) in the furosine treatment groups were expressed significantly higher than the control, suggesting that furosine activated the phosphoralation of *STAT1* and *STAT2* and expressions of *UBA7* and *UBE2L6*, which were the downstream sponsor of *STAT1* and *STAT2*, in a dosage-dependent manner. The above results further validated the effect of furosine in regulating these factors in *HepG2* cells.

Referring to cell apoptosis, the apoptotic cell rate was detected and analyzed by a flow cytometer, the effect of furosine in inducing cell apoptosis was primarily proved, and several related factors (*Caspase-3*, *Caspase-9*, *Bax*, and *Bcl-2*) were detected and discussed. *Caspase-3*, *Caspase-9*, and *Bax* are well-known enhancing factors, while *Bcl-2* is a classical apoptosis inhibitor [35,36,37]. These factors were measured before or after the treatment of furosine to evaluate the role of furosine in regulating downstream apoptosis. Results showed that furosine induced apoptosis of *HepG2* cells with a dosage-effect relationship. Furthermore, to investigate the effect of *STAT1*/*STAT2*/*UBA7*/*UBE2L6* on apoptosis induced by furosine, SiRNA fragments were transfected into the cells and apoptosis-related factors were detected. We found that after knocking down expressions of *STAT1*/*STAT2*/*UBA7*/*UBE2L6*, *Caspase-3*, *Caspase-9*, and *Bax* were down-regulated and *Bcl-2* was up-regulated significantly in furosine-treated groups, when compared with the control, indicating that *STAT1*/*STAT2*/*UBA7*/*UBE2L6* regulated expressions of the downstream apoptosis factors. The above results, for the first time, validated that furosine induced apoptosis through the regulation of *STAT1/2* and *UBA7/UBE2L6* genes in *HepG2* cells.

Considering that furosine could induced the apoptosis of liver cancer cells, its anti-tumor and anti-invasive effects in tumor-bearing animal models deserve our investigation and validation, which will be studied in the near future. Meanwhile, furosine was proved to be toxic to mice liver and kidney samples, so its toxicokinetics indicators and limit standard also deserve more attention and further research.

## 4. Materials and Methods

### 4.1. Chemicals 

Furosine was purchased from PolyPeptide (Strasbourg, France) with a purity of 95%. *HepG2* (liver hepatocellular cells) cell line was purchased from American Type Culture Collection Cells (ATCC, Manassas, VA, USA). Dulbecco’s Modified Eagle Medium (DMEM) and heat-inactivated fetal bovine serum (HI-FBS) were obtained from GIBCO (Waltham, MA, USA). l-glutamine was pruchased from Solarbio (Beijing, China). 1% penicillin/streptomycin was purchased from Thermo Fisher (Waltham, MA, USA). Cell counting kit-8 (CCK-8 kit) was purchased from Dojindo (Kumamoto, Japan). 0.1% (*v*/*v*) Triton X-100 and 100 mg/mL RNAase were purchased from Sigma (St. Louis, MO, USA). Triton X-100 staining solution was purchased from Roche (Berlin, Germany). RIPA lysate buffer (including PMSF and proteases) and the BCA detection kit were obtained from Beyotime (Shanghai, China). The TransZol Up Kit was purchased from TransGen Biotech (Beijing, China). Nanodrop 2000 was purchased from Thermo Fisher (Waltham, MA, USA). The High Capacity cDNA Archive Kit, Universal Master Mix, and RNAse-free water were purchased from Applied Biosystems (Foster City, CA, USA). All the primary antibodies and secondary antibodies were purchased from Santa Cruz (Santa Cruz, CA, USA). Protein lysis buffer and reagents related to western blotting were purchased from Solarbio (Beijing, China). Enhanced chemiluminescence (ECL) reagent was purchased from Tanon (Shanghai, China). The Annexin V/PI staining kit for cell apoptosis detection was purchased from Solarbio (Beijing, China).

### 4.2. Cell Culture and Viability Detection 

*HepG2* cells were cultured in DMEM containing 10% FBS, 0.9% l-glutamine, and 1% penicillin/streptomycin, in a humidified incubator (Thermo, Waltham, MA, USA) at 37 °C, in the presence of 5% CO_2_.

Cell viability was measured and analyzed by the CCK-8 kit assay. About 1 × 10^4^ cells per well were planted into 96-well plates and incubated for at least 24 h, and then different concentrations of furosine (the stock solution was 10 g/L, purified water as the solvent) ranging from 0–2000 mg/L in a total volume of 100 μL were added into the wells and cocultured for 48 h. Then, the medium was removed and 100 μL/well CCK-8 (Cell Counting Kit) solution was added into the wells for 4 h. Following this, the optical density (OD) value was detected by a microplate reader (Thermo, Waltham, MA, USA) at the wavelength of 570 nm. The proper dosage for further tests was chosen.

### 4.3. Transcriptomics Detection and Data Analysis

*HepG2* cells were cultured and treated with furosines (100/200 mg/L) for 48 h, and the cells were then gathered. Total RNA was extracted from the samples with the TRIzol™ Reagent (15,596,018, Invitrogen, Carlsbad, CA, USA) according to the manufacturer’s instructions. Extracted total RNA was quantified by Nanodrop2000 (Thermo Fisher Scientific, Waltham, MA, USA) and its quality was detected by the Bioanalyzer 2100 expert_RNA Nano 6000 Assay (Agilent, Santa Clara, CA, USA). mRNA isolation and the strand-specific mRNA-seq library preparation were performed using TruSeq Stranded mRNA Library Prep (20,020,594, Illumina, San Diego, CA, USA), including cDNA transcription, End repairing, A-tailing, and adapter ligation. Amplification was conducted for 15 cycles, followed by quality examination using the Bioanalyzer 2100 expert_High Sensitivity DNA Assay (Agilent) and Qubit™ 3 Fluorometer (Thermo Fisher Scientific). The libraries were then sequenced on the Illumina HiSeq platform (Supplementary Material). The data was exported into Excel spreadsheets by Simca-P for PCA (principle components analysis), PLS-DA (partial least squares discriminant analysis), *t*-test, and VIP (variable importance in projection) plot analysis (Supplementary Material).

### 4.4. Cell SiRNA Treatment

Based on primary analysis results in transcriptomics detection of *HepG2* cells, combined with the literature survey, *STAT1* (signal transducer and activator of transcription 1), *STAT2* (signal transducer and activator of transcription 2), *UBA7* (ubiquitin-like modifier activating enzyme 7), and *UBE2L6* (ubiquitin-conjugating enzyme E2L6) were chosen as the possible targets of furosine. To further validate the interaction between furosine and these factors, we synthesized their SiRNA sequences (genepharma, Shanghai, China). Cells were seeded into six-well plates for 24 h to ensure a 30% intensity. DNA-liposome complex was prepared as follows: (1) transfection reagent (4 μL/well, Santa Cruz) was diluted in 1 mL fresh DMEM; (2) SiRNA segments (4 μg/well) were diluted in 1 mL fresh DMEM; (3) the complexes of (1) and (2) were mixed together and DNA-liposome complex (2 mL per well) was added into the wells and these samples were placed at 37 °C. 24 h later, the DMEM-DNA-lipsome complex was added into DMEM to culture for another 24 h. The step of furosine treatment also started after removing the DMEM-DNA-lipsome complex, and the treatment time was 48 h. Utilizing western blotting detection of the aim proteins, the high-efficiency SiRNA fragments were screened out.

### 4.5. Quantitative Real-Time PCR (q-PCR) Analysis 

The total RNA was extracted from HepG2 cells using a TransZol Up Kit (Transgen, Beijing, China). The quantity and concentration of RNA were evaluated by 1.2% agarose gel electrophoresis and Nanodrop 2000 (Thermofisher, Waltham, MA, USA). The total RNA was transcribed into cDNA using a High Capacity cDNA Archive Kit, according to the manufacturer’s protocol. Primers for *STAT1*, *STAT2*, *UBA7*, *UBE2L6*, *Caspase-3*, *Bax*, *Bcl-2*, *Caspase-9*, and *GAPDH* are listed in Table 1, and *GAPDH* was chosen as the internal control. Quantitative real-time RT-PCR (qRT-PCR) was performed in 96-well plates in a total volume of 20 μL, containing 10 μL Universal Master Mix, 0.5 μL forward primer (10 μM), 0.5 μL reverse primer (10 μM), 1 μL template cDNA (cDNA, 10–15 ng/μL), and 8 μL RNAse-free water. All qRT-PCR reactions were performed at 94 °C for 30 s, followed by 40 cycles of 94 °C for 5 s, and 63 °C for 30 s, using two-step RT-PCR. All q-PCR reactions were performed on the ABI 7900 HT system, and were conducted in triplicate to ensure methodological reproducibility.

### 4.6. Western Blot Analysis

Total proteins of the cells (1 × 10^6^ cells per well) were extracted by lysis buffer containing protease inhibitors, and the sample was then centrifuged (4 °C, 12,000× *g*) for 5 min. With catalysis and heat treatment at 98 °C, the samples were loaded onto 12% SDS-polyacrylamide gels for electrophoresis and then transferred onto nitrocellulose filters by Trans-Blot machines (BioRad, Hercules, CA, USA). The filters were blocked with 2% BSA dissolved in TBST buffer for 2 h at room temperature. Then, the proteins were probed with the primary antibodies for 2 h at 25 °C, including *β-actin*, *STAT1*, *STAT2*, *P-STAT1*, *P-STAT2*, *UBA7*, *UBE2L6*, *Caspase-3*, *Bax*, *Bcl-2*, and *Caspase-9*. *β-actin* was regarded as the internal reference to promise equal loading. After the membranes were washed with PBST (phosphate buffered solution) buffer (10 min × 3), they were incubated with secondary antibodies for 2 h at room temperature and then washed (15 min × 3). Finally, the proteins were detected utilizing an ECL reagent (Millipore, Burlington, MA, USA) and analyzed by Image J software (Rawak Software, Inc., Stuttgart, Germany).

### 4.7. Immunofluorescence Cell Staining

For immunofluorescence cell staining, *HepG2* cells were seeded into the six-well plates (10% intensity). The cells were treated with furosine, and were then fixed, permeated with icy methanol for 20 min, washed thrice (15 min in total) with PBS, and blocked with 10% goat serum for 30 min. Primary antibody (1:250, Santa Cruz, CA, USA) was added and incubated for 1 h, and washed thrice (15 min in total) with PBS. Then, the cells were incubated for 30 min with the secondary antibodies (FITC-labelled, 1:500, Santa Cruz, CA, USA) and washed three times (5 min × 3). The nucleus was stained with PI (5 mg/mL as the final concentration, Sigma) for 15 min and washed (10 min × 3); the locations of *β-actin*, *STAT1*, *STAT2*, *UBA7*, and *UBE2L6* factors were scanned utilizing confocal microscopy (Zeiss, Oberkochen, Germany).

### 4.8. Cell Apoptosis Detection

After furosine treatment, cells were gathered and washed with PBS buffer, and were suspended in 200 μL binding buffer. The cell sample was incubated with 10 μL FITC-annexin V buffer and 20 μL PI buffer (10 mg/mL), then gently mixed together and incubated for 10 min at 25 °C in the dark. A total of 400 μL binding buffer was added into the cell samples and the samples were measured with flow cytometry immediately. Cells stained with annexin V−/PI+ stand for necrotic cells, annexin V+/PI+ cells stand for late apoptotic cells, and annexin V+/PI− cells stand for early apoptotic ones.

### 4.9. Statistical Analysis 

All the data was represented as Mean ± SD. Data analysis was performed using GraphPad Prism 6.0 software (GraphPad, San Diego, CA, USA). Statistical analysis was conducted using the Student’s *t*-test and One-Way Aanalysis of variance (ANOVA). In the experiments of cell viability, q-PCR, and western blotting, *p* value < 0.05 was considered to indicate a statistically significant difference between the control and furosine-treated groups.

## Figures and Tables

**Table 1:** Primers of the genes in q-PCR detection.

Gene Name	Primer Sequences (5′ → 3′)	
	Forward Primer	Reverse Primer
*STAT1*	GCTGCTGCTCCACAAGATGTT	TCTGCTGCCTTCGCTTCCA
*STAT2*	TGGCAGTGACAGAGGAGTTACA	CAGGCAATGGAGAGTTGGTTCA
*UBA7*	TCCAGAAGATGAGACGCTCCTT	AGCAACAGTCACACCTCCATTC
*UBE2L6*	GGAAGCCTTACACCAAGCCTTA	GAGTCAGGAGGTCAGCAAGTTC
*Caspase-3*	TGGAGGCTGACTTCCTGTATGC	TTCCGTTGCCACCTTCCTGTTA
*Bax*	CCAGGATGCGTCCACCAAGA	GAAGTCCAGTGTCCAGCCCAT
*Bcl-2*	GCATCTTCTCCTTCCAGCCTGA	TCTGCGAAGTCACGACGGTAG
*Caspase-9*	TGAATGACCACCTAGAGCCTTG	AGAACCACACCAGCCACAGT
*GAPDH*	CGTCCCGTAGACAAAATGGT	TTGATGGCAACAATCTCCAC

## Supplementary Materials

Supplementary materials can be found at http://www.mdpi.com/1422-0067/19/6/1629/s1.

## Abbreviations

HepG2	liver hepatocellular cells
STAT1	signal transducer and activator of transcription 1
STAT2	signal transducer and activator of transcription 2
UBA7	ubiquitin-like modifier activating enzyme 7
UBE2L6	ubiquitin-conjugating enzyme E2L6
